# Comparison of the Analgesic Effect of Ketamine and Midazolam with Paracetamol (Acetaminophen) and Ketorolac in Renal Colic Patients: A Clinical Trail: Comparison of the Ketamine and Midazolam with Paracetamol and Ketorolac

**DOI:** 10.31661/gmj.v14i.3593

**Published:** 2025-07-26

**Authors:** Anvar Bahrami, Latife Jabbari, Nahid Zamanimehr, Leila AzizKhani

**Affiliations:** ^1^ School of Medicine, Kurdistan University of Medical Sciences, Sanandaj, Iran; ^2^ Department of Emergency Medicine, Shahid Mohammadi Hospital, Hormozgan University of Medical Sciences, Bandar Abbas, Iran; ^3^ Department of Emergency Medicine, School of Medicine, Kurdistan University of Medical Sciences, Clinical Research Development Unit, Tohid Hospital, Kurdistan University of Medical Sciences, Kurdistan, Iran

**Keywords:** Ketorolac, Renal Colic, Ketamine, Acetaminophen, Midazolam, Pain Management

## Abstract

**Background:**

Renal colic is the most prevalent symptom of urinary stones, and it is quite painful. This study aimed to determine the effect of the Ketamine and Midazolam combination and compare it with the acetaminophen (paracetamol or Apotel) and Ketorolac (Toradol) combination in pain management of patients with renal colic in the emergency department (ED).

**Materials and Methods:**

In this double-blind clinical trial study, 200 renal colic patients admitted to the ED with more than 8 Numeric Rating Scale (NRS) of primary pain were divided into two groups by random blocking: one group received intravenous Ketamine (0.4 mg/kg), and intravenous Midazolam (at a dose of 0.016 mg/kg) and the other group received intravenous Ketorolac (30 mg) and intravenous acetaminophen (15 mg/kg). After that, we measured patients’ pain by NRS at 1, 5, 10, 15, 30, and 45 min after the procedure. The data were analyzed using IBM SPSS 21.0 software.

**Results:**

124 (62.0%) of 200 patients were men. Initial pain scores were 9(10-9) for Ketamine + Midazolam and 10(10-9) for Acetaminophen + Ketorolac.Linear regression was performed to compare the two groups’ adjusted pain scores, correcting for initial pain. The ultimate pain score increased by.392 units for each unit of starting pain. Group and time had significant effects (5.553, -.035, P=.001, respectively). Acetaminophen + Ketorolac had a higher mean pain score than Ketamine + Midazolam at all post-intervention time intervals. During the trial, both groups’ discomfort decreased significantly.

**Conclusion:**

The combination of Ketamine and Midazolam was more effective than Acetaminophen and Ketorolac in relieving the pain in renal colic. Therefore, if routine medications are contraindicated, a combination of Ketamine and Midazolam is recommended for pain control in patients with renal colic.

## Introduction

One of the most common reasons for emergency department (ED) visits is urinary stone pain, commonly known as renal colic [1]. Renal colic is the most prevalent symptom of urinary stones, and it is quite painful [2]. This problem affects approximately 1.2 million people in the United States each year and accounts for 1% of hospitalizations [1]. It is estimated that it affects 1-20% worldwide [3] and 1-5% of the population in affluent countries [4], causing discomfort to approximately millions of patients each year [3]. The expense of managing renal colic amounts to approximately £20 million, with patients typically spending a median of one day in the hospital [3]. Furthermore, prevalence of kidney stones in Iranian adult was reported to be 21% [3].

This is while its prevalence in Germany is between 4 and 4.7% [5]. therefore, effective and rapid treatments considered in emergency rooms are necessary.

According to international guidelines, non-steroidal anti-inflammatory drugs and opioids are the first choices for renal colic treatment (especially morphine, Ketorolac, and Acetaminophen); However, there are also drawbacks to using them, such as side effects and limits [6]. Non-steroidal anti-inflammatory drugs (NSAIDs) are the preferred pain reliever for people with renal colic. Compared to intramuscular (IM) injection or oral use, intravenous (IV) administration of these medications has a greater and faster effect.

On the other hand, these medications should be used with caution in individuals who are at risk of gastrointestinal bleeding or who have underlying renal issues. Furthermore, because NSAIDs cause platelet function abnormalities, they may cause lithotripsy to be delayed [7]. NSAIDs also interfere with the compensatory mechanisms of obstructed kidneys by reducing prostaglandins and may cause renal damage [8]. Narcotic medications like morphine and pethidine are another common treatment for people with acute renal colic. Despite their extensive use, these medications have several drawbacks, including side effects, a lack of public access, and the risk of addiction [9]. one of the researchers’ goals is to find an effective alternative drug with few side effects to first-line medications. Ketorolac (Toradol) is one of the NSAID labeled for intramuscular and intravenous administration for acute pain, and morphine is the best choice of opioids in renal colic [10][11]. Drug repeatedly used in research to confirm pain in the emergency department is intravenous acetaminophen (paracetamol or Apotel). This drug is recommended for the control of renal colic in patients, especially when non-steroidal anti- inflammatory drugs are contraindicated [12][13][14][15]. Another drug is Ketamine, which has anti-inflammatory properties in addition to anesthesia. It is also a pain reliever and has analgesic properties that distinguish it from other anesthetics. In addition, the side effects of this drug are minimal in analgesic doses [16][17][18]. Many physicians believe that agitation during recovery can be less by the concomitant use of Midazolam and Ketamine. Midazolam is a selective benzodiazepine used in anesthesia and has sedative and forgetfulness effects. when using this drug, there is a mismatch between the degree of sedation and forgetfulness, so that patients appear to be conscious but later do not remember the events of that time [19][20][21]. The combination of ketamine and midazolam offers significant advantages in pain management and sedation, including effective analgesia, reduced recovery agitation, and improved hemodynamic stability. Ketamine provides potent analgesia and dissociative anesthesia, while midazolam adds anxiolytic and amnesic effects, complementing ketamine’s profile. Studies show this combination reduces side effects compared to ketamine alone and is associated with better recovery profiles [22].

These benefits make it a compelling alternative to NSAIDs and opioids, particularly in settings where side effects or availability are concerns.

Because of the high pain intensity of renal colic patients, we need better and faster control of pain with fewer complications. We conducted this study to compare the effect of Ketamine and Midazolam with routinely used drugs in ED (Acetaminophen and Ketorolac) and achieve a combination with better efficacy and fewer side effects.

## Materials and Methods

This double-blind, randomized clinical trial was conducted from 1 April 2019 to 30 May 2020 in Kowsar Hospital, Sanandaj, Iran. The Ethics Committee of Kurdistan University of Medical Sciences approved the study’s protocol (approval number: IR.MUK.REC.1398.120.), and the study was registered in the Iranian Registry of Clinical Trials at www.irct.ir (registration code: IRCT20200422047163N1). This trial was carried out following the tenets of the Declaration of Helsinki. The objective and protocol of the study were explained to the subjects who met the inclusion criteria in simple language, and their informed consent was obtained in writing if they were willing to join the study. Participation in this research caused no disorder in diagnostic and therapeutic procedures, and no additional costs were imposed on the patients.

### Study Population

This study included patients over 18 to 65 years referred to the emergency department of Kowsar Hospital in 2019 and 2020. They had acute renal colic, clinical symptoms that suggested renal stones, and those with a history of renal calculus whose symptoms were comparable to past attacks and average initial pain according to the Numeric Rating Scale (NRS) system is greater than or equal to rank eight.

The NRS is a widely used tool for assessing pain intensity. It allows patients to rate their pain on a scale from 0 to 10, where 0 represents no pain and 10 indicates the worst imaginable pain. This scale is simple to administer, easy for patients to understand, and provides a standardized method for evaluating pain levels in clinical settings, making it a reliable tool for pain management studies [23].

The exclusion criteria were contraindications to the use of drugs, including schizophrenia, asthma, cardiovascular disease, Hypertension, Significant head trauma, Glaucoma, Pregnancy or suspected, Active lung infection, Hemodynamic instability, Active respiratory distress or hypoxemia, Acetaminophen allergy, Severe liver failure, Active liver disease, Active PUD (peptic ulcer disease) or history, Any suspicion of active bleeding, asthma, sensitivity to NSAIDs or aspirin and kidney disease, and not receiving painkillers (NSAIDs, Acetaminophen, opioids) in the last 4 hours.

Lack of cooperation in continuing the study, requests to leave the study by the patients and inability to understand the concept of the NRS chart are also the exclusion criteria of samples. We use the following equation to calculate the sample size in studies that aim to compare two means.


n = \frac{\left[2 \left(Z_{1 - \alpha/2} + Z_{1 - \beta} \right)\right]^2 \delta^2}{d^2}

With 5% alpha and 20% beta, the difference in standard deviation is equal to three tenths in both groups, and the lowest pain scores have a difference of one point (m1=3, m2=2). The sample size is calculated with the above formula in each group of 98 people. We consider 100 people in each group, so the total volume of the study is 200 people.

Randomization: Participants were randomly assigned to one of the two treatment groups (Ketamine + Midazolam or Acetaminophen + Ketorolac) using block random sampling. This method was employed to ensure balanced allocation of participants across the two groups. Blocks of a predefined size were used to randomly assign participants to each group, helping to maintain equal group sizes throughout the study.

Concealment of Randomization: To ensure that the allocation process was concealed from the researchers and participants, the randomization list was generated and maintained by a third-party coordinator who was not involved in the clinical intervention or outcome assessment. The allocation was kept in sealed, opaque envelopes that were opened only after the participant’s inclusion in the study.

**Figure-1:**
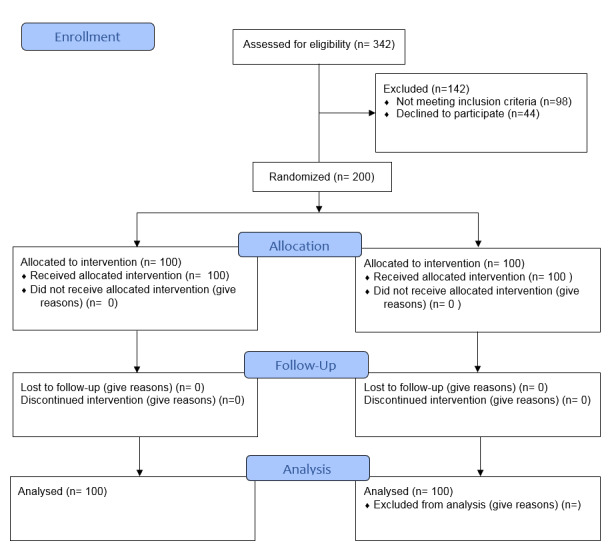
Figure 1. CONSORT flowchart of the participants of the study

Blinding: This study utilized a double-blind design. Both participants and the researchers who administered the treatments or assessed outcomes were unaware of group assignments. Blinding was maintained throughout the study to prevent bias in the administration of treatments and the evaluation of results (Figure-1).

### Intervention

After the approval of the Ethics Committee and IRCT, two hundred patients with renal colic patients referred to the emergency department of Kowsar Hospital in 2019 and 2020 were blocked by random blocking in two groups, A and B, which entered into the study in blocks of 10 by the physician according to the severity of pain based on the NRS chart.

After selecting the patients, their initial pain was recorded in the relevant checklist. The drug was then injected intravenously to match all patients for double blindness of study (due to the need to dilute Acetaminophen in serum). Serum therapy with normal saline in 500 ccs with a micro-set was done for all patients. Then the drugs were prescribed in such a way that for a group of Midazolam (Elixir Pharmaceutical Company (Boroujerd-Iran)) 0.016 mg/Kg of body weight and Ketamine (Ryan Drug Pharmaceutical Company) 0.4 mg/kg of body weight and for the second group Acetaminophen (Elixir Pharmaceutical Company) 15 mg/Kg of body weight and Ketorolac (Elixir Pharmaceutical Company) with a fixed dose of 30 mg was prescribed by a doctor. Then the patient’s pain is scored according to the NRS system after the medication is prescribed. First, immediately after drug injection and then at the specified times, one minute, five minutes, ten minutes, fifteen minutes, thirty minutes, and forty-five minutes after drug injection. Complications of medications (allergies, apnea, respiratory disorders, hallucinations) were assessed simultaneously

If the patient’s pain was unbearable for the patient after receiving the drug and reaching the peak effect or during the study, Fentanyl (from Abu Reihan Pharmaceutical Company) was used to control the pain with a dose of one microgram/kilogram of body weight. The criterion for improving patients’ pain is a score of less than three on the NRS nomogram. Ketamine was injected in a low dose (sub dissociative) in this study, and the patient was under cardiac and respiratory monitoring during the study. Ketamine injection was performed under the supervision of an emergency medicine specialist to take the necessary action in case of a severe respiratory complication. Patients were evaluated for the presence or absence of urinary stones after the pain was controlled and the general condition stabilized based on the ultrasound results. If there were no urinary stones on the ultrasound, they were CT scanner, and If Scans also showed no urinary stones, patients were excluded from the study. The information was recorded in a checklist and prepared for statistical analysis.

### Statistical Analysis

The data were analyzed using IBM SPSS Statistics for Windows, version 21 (IBM Corp., Armonk, N.Y., USA). The continuous variables are described with median (interquartile range), and the categorical variables are expressed as frequencies (percentages). The normality of the data was assessed using the one sample Kolmogorov-Smirnov test, therefore, Chi-square, Fisher exact test, and Mann-Whitney U test were used to compare the baseline characteristics and pain intensity during the time between groups. Adjusted Linear regression was used to compare the pain score between the two groups using the Generalized Estimating Equation (GEE), considering the outcome variable’s repeated measures. The generalized linear mixed model (GLMM) was used to analyze the data. This method was chosen because of the presence of repeated measures at seven different times (Time0, Time1, Time5, Time10, Time15, Time30, Time45) and the comparison of two intervention and control groups. In this model, time was considered as a within-subject factor and group as a between-subject factor. Main and interaction effects between time and group were examined.

P-value lower than 0.05 was considered as significant level.

### Ethical Approval

The authors of the study certify that the study was performed in accordance with the ethical standards as laid down in the 1964 Declaration of Helsinki and its later amendments or comparable ethical standards and was approved by the council of the Ethics Committee of Kurdistan University of Medical Sciences under the number IR.MUK.REC.1398.120.

## Results

**Table:** Table1. Baseline Characteristics, Comparison between Ketamine + Midazolam and Acetaminophen + Ketorolac Groups

Variable		Ketamine + Midazolam N = 100	Acetaminophen + Ketorolac N = 100	P-value
Gender	Male	64(64.0%)	60(60.0%)	0.560
Age	(mean, SD)	35(30-41, SD: 11.4)	36(28-45, SD: 11.6)	0.888
Stone Size	(mean, SD)	6.0(5.0-8.0, SD: 2.6)	6.0(4.8-8.0, SD: 1.9)	0.705
Complication	No	96 (96.0%)	100 (100.0%)	0.121
	Yes: hallucinations	4(4.0%)	0(0.0%)	
	1	1(1.0%)	10(10.0%)	
fentanyl	2	99(99.0%)	89(89.0%)	0.005
	9	0(0.0%)	1(1.0%)	

SD: standard deviation

**Table:** Table2. Comparing pain Scores in Ketamine/Midazolam and Acetaminophen/Ketorolac Groups by Multiple Regression and Generalized Estimated Equation Method

Variable	Coefficient	95% CI	P-value
Initial Pain	.392	(.184-.599)	<0.001
Group Acetaminophen+Ketorolac Ketamine+Midazolam	5.553 Reff	(5.316 -5.790) Reff	<0.001
Time	-.035	(-.040 -.030)	<0.001
Group * Time	-.114	(-.124 -.104)	<0.001

Of 200 patients included in the study, 124 (62.0%) were men. The two groups were homogeneous in terms of baseline variables (i.e. (Gender, Age, and Stone Size). Baseline characteristics, Comparison between Ketamine + Midazolam and Acetaminophen + Ketorolac groups were indicated in Table-1.

The media of initial pain intensity scores were 9(10-9) and 10(10-9) in the Ketamine + Midazolam and Acetaminophen + Ketorolac groups, respectively (P=.027, Table-2).

Analysis using a generalized linear mixed model (GLMM) showed that the main effect of time on pain intensity was significant (F (6, 294)=15.32, P<0.001), meaning that pain intensity changed significantly over time. The main effect of group was also significant (F(1, 49)=8.45, P=0.005), such that the intervention group showed a greater reduction in pain intensity than the control group.

The interaction effect of time and group was also significant (F (6, 294)=12.87, P<0.001), indicating that changes in pain intensity over time were different between the two groups. These results indicate a strong effect of the intervention in reducing pain compared to the control group.

Furthermore, linear regression analysis assessed the adjusted pain score between the two groups, controlling initial pain effects. By increasing one unit in the initial pain score, the final pain score increased by .392 units. The effect of group and time was statistically significant (5.553 P-value<.001, -.035, P-value<.001), respectively.

**Figure-2:**
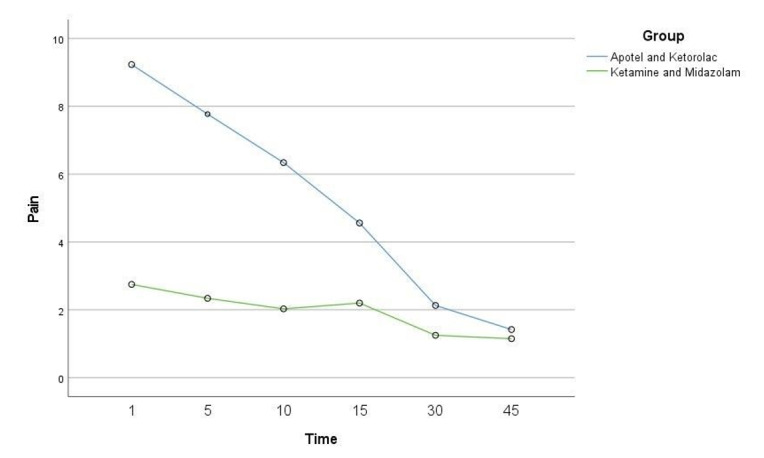
Figure 2. Plot of two group displaying the change in pain intensity from baseline to 45 min

On the other hand, the interaction of time and groups were significant; accordingly, the severity of the pain decreased over time. The mean difference in pain at times one, five, ten, fifteen, thirty, and forty-five in the Ketamine+Midazolam group was 5.43 (SD: 0.93), 4.98(SD: 1.27), 4.41(SD: 1.34), 3.84(SD: 1.67), 2.13(SD: 1.93), and 0.42(SD: 1.91) less than Acetaminophen+Ketorolac group. Overall, the mean pain score was higher in the Acetaminophen+Ketorolac than the Ketamine+Midazolam group at all post-intervention time points (Table-3). Nevertheless, the pain intensity significantly and consecutively reduced in both groups during the study (Figure-2).

## Discussion

**Table:** Table3. Pain Intensity Changes at Different Times in Ketamine + Midazolam and Acetaminophen + Ketorolac Groups

Variable	Ketamine + Midazolam N = 100	Acetaminophen + Ketorolac N = 100	P-value
Initial Pain	9(10-9, SD: 1.1)	10(10-9, SD: 0.8)	0.027
Pain in Time 1	3(4-2, SD: 0.9)	9(10-9, SD: 0.9)	<0.001
Pain in Time 5	2(3-2, SD: 0.9)	8(9-7, SD: 1.5)	<0.001
Pain in Time 10	2(2-1, SD: 0.8)	6(8-5, SD: 1.7)	<0.001
Pain in Time 15	2(3-1, SD: 1.1)	4(6-3, SD: 2.1)	<0.001
Pain in Time 30	1(2-1, SD: 0.9)	1(3-0, SD: 2.6)	0.239
Pain in Time 45	0(1-0, SD: 0.9)	1(2-0, SD: 2.5)	0.007

The results of this study demonstrate that the combination of Ketamine and Midazolam is more effective in reducing pain intensity in renal colic patients compared to the Acetaminophen and Ketorolac combination. Both groups were homogenous in baseline variables, including gender, age, and stone size, which strengthens the validity of our findings. In the present study, the comparison of the groups in terms of pain intensity at different times showed that the pain intensity was similar in the two groups before the injection of drugs. There was no significant difference. However, in other study times (1, 5, 10, 15, 30 and 45 minutes) after drug injection, there was a significant difference between the two groups, so the Ketamine + Midazolam group showed a significantly more significant pain reduction than the Acetaminophen + ketorolac group. Also, the need for Fentanyl to control pain was significantly lower in the ketamine + midazolam group (0%) than in the Acetaminophen + ketorolac group (11%). The initial pain intensity scores revealed a slight but statistically significant difference between the two groups, with the Ketamine + Midazolam group having a lower median score than the Acetaminophen + Ketorolac group. This difference was accounted for in subsequent analyses using generalized linear mixed models (GLMM), ensuring that the comparison of analgesic effects was adjusted for baseline differences.

Several studies have been performed on the effect of Ketamine on various types of pain, but studies on the effect of intravenous Ketamine in renal colic are limited. In this regard, Sotoodehnia et al [7]. In a double- blind clinical trial study, 126 renal colic patients referred to the emergency department were randomly divided into low-dose ketamine (0.6 mg/kg body weight) and intravenous ketorolac (30 mg) recipients. The results showed that there was no significant difference between the two groups at other times (15, 30, 60, 120), except for 5 minutes after drug injection (with greater effectiveness of Ketamine [7]. Despite higher doses of Ketamine and equal doses of Ketorolac compared to our study, except for 5 minutes after drug injection, there was no significant difference between Sotoodehnia et al. groups. A reason may be due to the effect of Midazolam. A study conducted by Fatemeh Safi et al.; reported that Midazolam has a good effect on pain relief during and after hysterosalpingography in women with infertility. As a result, Midazolam, apart from reducing the side effects of Ketamine, can have an auxiliary effect on pain relief in renal colic patients [24][25].

The results of a review study conducted in 2013 by Person J demonstrate the effect Ketamine has on postoperative pain, specifically its effect on burn pain in patients [26].

Morphine and pethidine are other common treatments for people with acute renal colic, especially when the pain is not relieved with common drugs. Despite their extensive use, these medications have some drawbacks, including side effects, a lack of public access, and the risk of addiction, so we need to decrease their use [9]. In our Ketamine + midazolam group study, decreasing need for this drug was seen. In a review study by Sabramaniam K et al., the Ketamine effect on pain after surgery was evaluated and showed that patients who received Ketamine had less pain and less need for opioids. low doses of Ketamine could be helpful and safe in routine opioid analgesic procedures [27].

In another study conducted by Forouzan et al. [28], 135 renal colic patients referred to the emergency department with initial pain greater than five according to NRS, into two groups receiving low-dose intravenous Ketamine (0.3 mg/kg body weight) and intravenous morphine (0.1 mg/kg body weight) were randomly divided. Except for 30 minutes after drug injection (with greater efficacy of Ketamine), at other times (10, 20, 60 minutes), no significant difference was observed between the two groups and in the Morphine recipients group, reported a significant difference in increasing the need for Fentanyl for pain control compared with the ketamine group. the results showed that Ketamine could compete with narcotics in painkilling, and also it can decrease the need for Fentanyl. a limited number of previous studies have shown different results from our study. In a double-blind clinical trial conducted by Vosoughin et al. 80 female patients undergoing gynecological laparoscopic surgery were randomly divided into two groups: one was receiving Intravenous Ketamine (0.15 mg/kg body weight) and the other group receiving rectal diclofenac suppository (100 mg) and evaluated for postoperative pain intensity. The intensity of pain and need for morphine at 1, 3, and 6 hours (between 1, 3, 6, and 12 hours) after surgery was lower than in the ketamine group. It showed that diclofenac suppository 100 mg was more effective than intravenous Ketamine in pain control after laparoscopic gynecological surgery [29]. these differences may be due to the number of patients studied. The difference in the dose of Ketamine used the characteristics of patients, including underlying diseases, addiction, and their previous use of painkillers, The pharmaceutical company of the drug and the addition of Midazolam to Ketamine.

Other results showed that side effects in the group receiving Ketamine + Midazolam were not significantly different from the Acetaminophen + Ketorolac group. Four hallucinations were reported in the ketamine + midazolam group, and other complications (apnea, Respiratory disturbance, and allergies) were not observed in any group. In this regard, a prospective study by Tran KP et al., Which compared the analgesic effects of Ketamine and intravenous morphine in the care of trauma patients before hospitalization, showed that the analgesic effects of Ketamine and morphine were similar with more hallucinations and restlessness in the ketamine group. He received more Ketamine than the other group (11% vs. 1.5%) [30]. This may be due to the lack of Midazolam in this study. A review study by Murcia et al. the effect of Ketamine on postoperative pain in 2024, a few studies showed that patients receiving Ketamine without benzodiazepines are more likely to have hallucinations [31] And in a study by Sener S et al. On emergency sedation of patients Through Ketamine with and without Midazolam, it was shown that adding Midazolam to Ketamine significantly reduced restlessness during recovery after ketamine injection [32].

The clinical implications of these findings are substantial. Ketamine + Midazolam may serve as a valuable alternative for patients with contraindications to first-line analgesics such as opioids, NSAIDs, or Acetaminophen. Additionally, its rapid and sustained analgesic effects make it a promising option in acute pain management settings where prompt relief is critical.

This study has several limitations that should be considered. First, the relatively short follow-up period limits the assessment of long-term outcomes and potential adverse effects of the interventions. Second, the single-center design may affect the generalizability of the results to other settings. Future studies with larger, multicenter populations and extended follow-up periods are recommended to confirm these findings and explore additional applications of Ketamine + Midazolam in pain management.

## Conclusion

According to the present study, the effect of ketamine + midazolam combination in controlling pain in renal colic patients and reducing the need for Fentanyl and other analgesics is more than the Acetaminophen- ketorolac combination. However, in contraindications to First-line and common drugs (Ketorolac, opioid, Acetaminophen), Ketamine + Midazolam combination can be considered. Future studies could further investigate the long-term safety and efficacy of this combination in various clinical settings, as well as explore its potential use in other pain management scenarios. Among the limitations of the study, the short follow-up period and relatively low generalizability of the results may be occurred.

## Conflict of Interests

The authors declare that they have no competing interest.

## References

[R1] Wang X, Cao Y, Hu J, Jia L-C, Li B, Liu B (2024). Effect of Early-Intervention Acupuncture on Pain Relief Among
Emergency Department Patients with Suspected Acute Renal Colic Due to
Urinary Calculi: Study Protocol for a Randomized Clinical Trial. Journal of Pain Research.

[R2] Abdulrasheed H, Adenipekun A, Mohsin MS, Khattak MA, Elsayed W, Cheema H (2024). Audit of the Acute Management of Renal Colic in District
Hospitals Within a National Health Service Trust. Cureus.

[R3] Qu Z, Wang T, Tu J, Yao W, Pei X, Jia L (2022). Efficacy and Safety of Acupuncture in Renal Colic Caused by
Urinary Calculi in Adults: A Systematic Review and Meta-Analysis. Evidence-Based Complementary and Alternative Medicine.

[R4] Bhattacharya S, Joshi NK, Jain YK, Bajpai N, Bhardwaj P, Chaturvedi M (2022). Dietary Determinants of Renal Calculi: A Case-Control Study From
a Tertiary Care Hospital of Western Rajasthan. Cureus.

[R5] Stamatelou K, Goldfarb DS (2023). Epidemiology of Kidney Stones. Healthcare.

[R6] Lee JY, Andonian S, Bhojani N, Bjazevic J, Chew BH, De S (2021). Canadian Urological Association guideline: Management of ureteral
calculi–Full-text. Canadian Urological Association Journal.

[R7] Sotoodehnia M, Farmahini-Farahani M, Safaie A, Rasooli F, Baratloo A (2019). Low-dose intravenous ketamine versus intravenous ketorolac in
pain control in patients with acute renal colic in an emergency setting: a
double-blind randomized clinical trial. Korean J Pain.

[R8] Klomjit N, Ungprasert P (2022). Acute kidney injury associated with non-steroidal
anti-inflammatory drugs. European Journal of Internal Medicine.

[R9] Morteza-Bagi HR, Amjadi M, Mirzaii-Sousefidi R (2015). The Comparison of Apotel plus Low Dose of Morphine and Full Dose
of Morphine in Pain ‎Relief in Patients with Acute Renal Colic. Addiction & Health.

[R10] Seyhan AU, Yılmaz E (2021). Treatment of Renal Colic by Nerve Blockade with Lidocaine Versus
Intravenous Dexketoprofen. Age.

[R11] Eskew J, Kelly T, Ode G (2023). Ketorolac as a Local Analgesic in Orthopaedic Conditions: A
Systematic Review of Safety and Efficacy. Current Orthopaedic Practice.

[R12] Jaballah R, Toumia M, Youssef R, Ali KBH, Bakir A, Sassi S (2025). Piroxicam and paracetamol in the prevention of early recurrent
pain and emergency department readmission after renal colic: Randomized
placebo-controlled trial. Academic Emergency Medicine.

[R13] Thia I, Saluja M (2021). An update on management of renal colic. Australian Journal of General Practice.

[R14] Nasar AA, Ahmad A, Saleem S, Bary A, Sarfraz J, Amjad M (2024). Efficacy of IV Paracetamol in Patients with Renal Colic in
Emergency Department. Indus Journal of Bioscience Research.

[R15] Freo U, Ruocco C, Valerio A, Scagnol I, Nisoli E (2021). Paracetamol: a review of guideline recommendations. Journal of clinical medicine.

[R16] Simonini A, Brogi E, Cascella M, Vittori A (2022). Advantages of ketamine in pediatric anesthesia. Open Medicine.

[R17] Natoli S (2021). The multiple faces of ketamine in anaesthesia and analgesia. Drugs Context.

[R18] Hirota K, Lambert DG (2022). Ketamine; history and role in anesthetic pharmacology. Neuropharmacology.

[R19] Peter J-U, Dieudonné P, Zolk O (2024). Pharmacokinetics, Pharmacodynamics, and Side Effects of
Midazolam: A Review and Case Example. Pharmaceuticals.

[R20] Rao A, Tiwari S (2024). Understanding Midazolam: The Key to Its Safe Clinical Use
Midazolam in Pediatric Dentistry. Springer.

[R21] Noor N, Legendre R, Cloutet A, Chitneni A, Varrassi G, Kaye AD (2021). A comprehensive review of remimazolam for sedation. Health psychology research.

[R22] Khan MT, Khan AR, Rohail S, Raza FA, Ahmed S, Siddiqui A (2024). Safety of procedural sedation in emergency department settings
among the adult population: a systematic review and meta-analysis of
randomized controlled trials. Internal and Emergency Medicine.

[R23] Nugent SM, Lovejoy TI, Shull S, Dobscha SK, Morasco BJ (2021). Associations of Pain Numeric Rating Scale Scores Collected during
Usual Care with Research Administered Patient Reported Pain Outcomes. Pain Med.

[R24] Laskowski K, Stirling A, McKay WP, Lim HJ (2011). A systematic review of intravenous ketamine for postoperative
analgesia. Can J Anaesth.

[R25] Safi F, Rabiee L, Shokrpour M, Kamali A (2019). Comparison of midazolam and dexmedetomidine for pain relief
during and after hysterosalpingography in women with infertility. J Med Life.

[R26] Persson J (2013). Ketamine in pain management. CNS Neurosci Ther.

[R27] Reede K, Bartholomew R, Nielsen D, Ahmeti M, Zreik K (2023). Ketamine in Trauma: A Literature Review and Administration
Guidelines. Cureus.

[R28] Forouzan A, Masoumi K, Motamed H, Esfahani SRN, Delirrooyfard A (2019). Comparison of the Analgesic Effect of Intravenous Ketamine versus
Intravenous Morphine in Reducing Pain of Renal Colic Patients: Double-Blind
Clinical Trial Study. Rev Recent Clin Trials.

[R29] Vosoughin M, Mohammadi S, Dabbagh A (2012). Intravenous ketamine compared with diclofenac suppository in
suppressing acute postoperative pain in women undergoing gynecologic
laparoscopy. J Anesth.

[R30] Tran KP, Nguyen Q, Truong XN, Le V, Le VP, Mai N (2014). A comparison of ketamine and morphine analgesia in prehospital
trauma care: a cluster randomized clinical trial in rural Quang Tri
province, Vietnam. Prehosp Emerg Care.

[R31] Kerguelen Murcia, Li JS, Phatak UR (2024). Impact of intra-operative ketamine on postoperative outcomes in
abdominal surgery: a narrative review. Translational Gastroenterology and Hepatology.

[R32] Sener S, Eken C, Schultz CH, Serinken M, Ozsarac M (2011). Ketamine with and without midazolam for emergency department sedation in adults: a randomized controlled trial. Ann Emerg Med.

